# Implications of multimorbidity patterns on health care utilisation and quality of life in middle-income countries: cross-sectional analysis

**DOI:** 10.7189/jogh.09.020413

**Published:** 2019-12

**Authors:** Grace Sum, Chris Salisbury, Gerald Choon-Huat Koh, Rifat Atun, Brian Oldenburg, Barbara McPake, Sukumar Vellakkal, John Tayu Lee

**Affiliations:** 1 Saw Swee Hock School of Public Health, National University of Singapore, Singapore; 2 Centre for Academic Primary Care, NIHR School for Primary Care Research, Population Health Sciences, Bristol Medical School, University of Bristol, Bristol, UK; 3 Harvard T.H Chan, School of Public Health, and Harvard Medical School, Harvard University, Cambridge, Massachusetts, USA; 4 Nossal Institute for Global Health, Melbourne School of Population and Global Health, University of Melbourne, Australia; 5 WHO Collaborating Centre on Implementation Research for Prevention & Control of NCDs; 6 Birla Institute of Technology and Science, Pilani, K. K. Birla Goa Campus, Goa, India; 7 Public Health Policy Evaluation Unit, School of Public Health, Imperial College London, UK

## Abstract

**Background:**

Past studies have demonstrated how single non-communicable diseases (NCDs) affect health care utilisation and quality of life (QoL), but not how different NCD combinations interact to affect these. Our study aims to investigate the prevalence of NCD dyad and triad combinations, and the implications of different NCD dyad combinations on health care utilisation and QoL.

**Methods:**

Our study utilised cross-sectional data from the WHO SAGE study to examine the most prevalent NCD combinations in six large middle-income countries (MICs). Subjects were mostly aged 50 years and above, with a smaller proportion aged 18 to 49 years. Multivariable linear regression was applied to investigate which NCD dyads increased or decreased health care utilisation and QoL, compared with subjects with only one NCD.

**Results:**

The study included 41 557 subjects. Most prevalent NCD combinations differed by subgroups, including age, gender, income, and residence (urban vs rural). Diabetes, stroke, and depression had the largest effect on increasing mean number of outpatient visits, increasing mean number of hospitalisation days, and decreasing mean QoL scores, respectively. Out of the 36 NCD dyads in our study, thirteen, four, and five dyad combinations were associated with higher or lower mean number of outpatient visits, mean number of hospitalisations, or mean QoL scores, respectively, compared with treating separate patients with one NCD each. Dyads of depression were associated with fewer mean outpatient visits, more hospitalisations, and lower mean QoL scores, compared to patients with one NCD. Dyads of hypertension and diabetes were also associated with a reduced mean number of outpatient visits.

**Conclusions:**

Certain NCD combinations increase or decrease health care utilisation and QoL substantially more than treating separate patients with one NCD each. Health systems should consider the needs of patients with different multimorbidity patterns to effectively respond to the demands on health care utilisation and to mitigate adverse effects on QoL.

Rapid demographic transition has led to an ageing population in middle-income countries (MICs) [1,2], with a rising burden of non-communicable diseases (NCDs) including patients with multimorbidity, the presence of two or more NCDs [3]. Equitable health care access to meet the needs of patients with multimorbidity who experience diminished quality of life (QoL) is a major challenge for health systems in MICs [4,5].

Studies in MICs have shown positive associations between the presence of NCDs in patients and their health care utilisation, and an inverse relationship between NCDs and QoL [6,7]. However, these studies have focused on single NCDs, or specific pairs of comorbid conditions, often with hypertension or diabetes as the index conditions [8-10]. These findings cannot be generalised to multimorbidity, as the simultaneous presence of multiple conditions may not only have an additive effect, but could also work synergistically to have a disproportionate adverse impact on health care utilisation, health spending, and QoL [11,12]. Conversely, some combinations of conditions may lead to benefits such as reduced health service utilisation compared to one condition, when care for one NCD is jointly provided with another. A UK study on NCD combinations suggested that certain NCD combinations led to increased or decreased health care cost, compared with treating separate patients that have one NCD [11]. This study found that depression was associated with increased costs, when co-occurring with other NCDs. Although dementia was cost-limiting for health care, it was cost-increasing when social care expenditure was included [11].

This is the first study that explores the impact of multimorbid combinations in six MICs with large sized populations. It investigates the prevalence of combinations of nine important NCDs and the impact of these combinations on health care utilisation and QoL, compared with treating separate patients with one NCD. Our study aims to provide a better understanding of how NCDs interact to produce different patterns of multimorbidity with important implications for health policy (workforce/capacity planning; resource allocation), and clinical management. Examination of patterns of multimorbidity also informs the design of interventions to meet the needs of patients with multimorbidity.

## METHODS

### Sample and data

We used cross-sectional data from the World Health Organisation (WHO) Study of Global Ageing and Adult Health (SAGE) Wave 1 (2007-10). This provides nationally representative samples of individuals aged 18+ years in China, Ghana, India, Mexico, Russia and South Africa [13]. The WHO sampling methodology ensures that the samples in each of the six countries are nationally representative, and includes a sample of respondents from SAGE Wave 0 (2002-2004) in the six countries with new respondents added to SAGE Wave 1 [14].

The aim of SAGE is to generate valid, reliable, and comparable information on a range of health and well-being outcomes of public health importance. SAGE is internationally recognised as one of the highest quality sources to examine health outcomes in MICs [14]. The participating SAGE countries are from different geographic areas, have different levels of economic development, and are at different stages in demographic and health transition. SAGE includes two countries, China and India, with the largest populations in the world [13].

The original sample size in the six study countries was 47 443 (China: 15 050, India: 12 198, Ghana: 5573, Russia: 4947, Mexico: 5448, South Africa: 4227). After excluding adult subjects who failed to indicate their age, our total sample size was 44 089 (China: 15 009, India: 12 198, Ghana: 5563, Russia: 4350, Mexico: 2744, South Africa: 4225). We excluded those who had missing values on outcome variables and covariates (5.7% of entire sample). The final sample size was 41 557 (China: 14 906, India: 11 159, Ghana: 5067, Russia: 4330, Mexico: 2618, South Africa: 3477). Figure S1 in **Online Supplementary Document** shows flowcharts summarising the data-cleaning process.

### Variables

#### Non-communicable disease ascertainment

SAGE includes nine NCDs: angina, arthritis, asthma, cataracts, diabetes, stroke, chronic lung disease, hypertension and depression.

Subjects were defined as having the NCD by self-reported diagnoses, or symptom-based assessment by SAGE survey, or both.

In line with previous studies, we defined respondents as self-reporting an NCD if they answered affirmatively to: “Have you ever been diagnosed with…?” [13,15,16].

Hypertension was derived from self-reported diagnosis, or the average of three physical measurements of blood pressure (systolic blood pressure ≥140mmHg or diastolic blood pressure ≥90mmHg), or both.

Five of the nine NCDs (angina, arthritis, asthma, chronic lung disease, and depression) were derived from self-reported diagnosis, or symptom-based assessment, or both. Symptom-based assessments were based on validated symptom scales derived through a standard algorithm based on a set of symptomatic questions from the SAGE survey for each of the diseases (eg, Rose questionnaire for angina [17,18]. Composite International Diagnostic Interview for depression [19,20], receiver operating characteristic curve analysis that generated an algorithm for arthritis diagnosis by symptoms [21]).

Three of the nine NCDs, diabetes, stroke, and cataract were derived from self-reported diagnosis only.

These methods are consistent with SAGE individual country reports published by WHO [15,22]. Table S1 in **Online Supplementary Document** lists algorithms to ascertain the presence of NCDs.

#### Predicting and outcome variables

We examined the prevalence of NCD dyad and triad combinations. Subjects with an NCD dyad, for example, depression + angina, were those who have at least those two NCDs. Similarly, subjects with a NCD triad are those who have at least those three NCDs.

Our analyses to predict the impact of combinations of NCDs on health care utilisation and QoL were based on the 36 NCD dyads generated by the nine NCDs in SAGE. The number of triad combinations would have been unfeasibly large for analysis and difficult to interpret.

We stratified subjects according to three age groups, including those aged 18-49 years (young adults), 50-64 years, and 65+ years (elderly).

Outcome variables were health care utilisation and QoL. Health care utilisation referred to outpatient care and hospitalisations. Respondents were asked about the number of times they had outpatient visits in the last 12 months, and the number of overnight stays in the hospital that lasted for at least one night, in the last 12 months. The QoL score was measured by an 8-item WHO Quality of Life (WHOQoL) instrument in SAGE.[23] The WHOQoL included two questions in each of four broad domains: physical, psychological, social, and environmental. Respondents rated their satisfaction with different domains of their lives, such as money, health, and relationships, as well as rating their overall life satisfaction using a five-point response scale, ranging from very satisfied to very dissatisfied. A composite score using these 8 items was created, by summing the responses across these 8-items, and rescaling the result from 0–100 where a higher score indicated better QoL [23].

#### Covariates

Age, gender, marital status, education (primary or less, secondary, tertiary and above), individual’s income quintile, residence (rural/urban), health insurance (with/without insurance), and country.

### Statistical analysis

We pooled data from all six MICs and stratified most of the findings by age (18-49 years, 50-64 years, ≥65 years). We summarised subject characteristics by country, and by multimorbidity status. We analysed the prevalence of subjects for each of the nine individual NCDs, and NCD dyad and triad combinations. We presented the most common dyads and triads using a cut-off prevalence of 1.5%. We examined the most common NCD dyads by age, gender, income, and residence (urban vs rural). We assessed associations between each of the nine individual NCDs, health care utilisation and QoL, using multivariable linear regression.

We studied the interaction (effect modification) of NCD dyads on health care utilisation and QoL, using multivariable linear regression models. Each multivariable model included an interaction term for one of the NCD dyads, each of the nine individual NCDs, and covariates.

Statistically significant positive interaction terms indicated that the NCD dyads were utilisation-increasing or QoL-increasing, while statistically significant negative interaction terms indicated that the NCD dyads were utilisation-decreasing or QoL-decreasing. Non-statistically significant interaction terms indicated that NCD dyads were utilisation-neutral or QoL-neutral. We presented regression coefficients and *P* values for multivariable linear regression models for the mean number of outpatient visits, mean number of hospitalisations, and mean QoL scores.

We tested multicollinearity for covariates to find that the multicollinearity diagnostics (Variance Inflation Factor) were less than five, indicating that the assumption of reasonable independence among predictor variables was met [24]. Analyses were weighted by sample size and country dummy variables, to account for the complex multi-stage design of SAGE. We performed statistical analyses using Stata 15∙1(StataCorp). Statistical significance was set at *P* < 0.05.

## RESULTS

### Sample characteristics

In **Table 1** we present the socio-demographic characteristics for the 41 557 adults aged ≥18 years by multimorbidity status, and by country in Table S2 in **Online Supplementary Document**. Median age was 58 (IQR = 51-68) years. Patients with multimorbidity were more likely to be female, unmarried, in the lowest income quintile, in urban areas, and with insurance.

**Table 1:** Subject characteristics by multimorbidity status, using pooled data

Multimorbidity status (n = 41 557)							
	**0 NCDs**	**1 NCD**	**2 NCDs**	**3 NCDs**	**4 NCDs**	**5 NCDs**	**≥6 NCDs**
**N (row %)**	26 600 (64.01)	7077 (17.03)	3711 (8.93)	2102 (5.06)	1167 (2.81)	511 (1.23)	394 (0.95)
**Gender (%):**							
Male	49.19	56.38	45.18	35.82	44.03	50.49	43.20
Female	50.81	43.62	54.82	64.18	55.97	49.51	56.80
**Marital status (%):**							
Not married	21.19	16.35	22.50	34.23	27.56	38.32	43.95
Married	78.81	83.65	77.50	65.77	72.44	61.68	56.05
**Age (mean, in years)**	41.48	45.82	54.18	58.35	61.33	62.07	61.82
**Education level (%):**							
No schooling	18.22	13.44	18.32	20.94	18.36	18.61	23.26
Primary or lower	22.48	26.60	24.44	23.69	21.00	18.93	11.58
Secondary	21.95	25.65	19.12	19.79	14.24	21.13	15.77
Tertiary or higher	37.35	34.31	38.11	35.58	46.40	41.33	49.40
**Wealth quintile (%):**							
Q1 (lowest)	15.24	13.20	11.82	18.10	13.08	12.78	19.03
Q2	18.03	17.10	18.41	18.14	17.17	22.58	12.90
Q3	18.81	18.91	17.79	19.77	20.96	20.39	37.88
Q4	20.12	23.29	26.37	20.69	23.46	23.07	12.53
Q5 (highest)	27.79	27.50	25.61	23.29	25.34	21.18	17.66
**Location (%)**							
Rural	56.08	54.60	50.80	47.44	49.09	39.11	38.32
Urban	43.92	45.40	49.20	52.56	50.91	60.98	61.68
**Insurance (%)**							
No insurance	72.95	66.22	47.75	44.63	35.95	43.25	39.70
With insurance (mandatory/voluntary)	27.05	33.78	52.25	55.37	64.05	56.75	60.30

NCD – non-communicable disease

Figure S2 in **Online Supplementary Document** shows the prevalence of single NCDs stratified by age.

### Non-communicable disease dyad and triad prevalence

**Figure 1:**
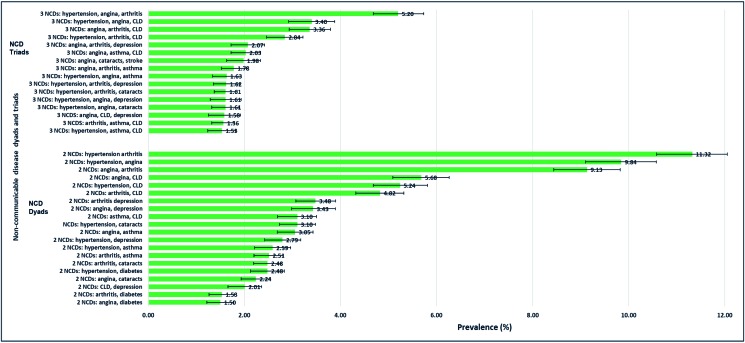
Most prevalent non-communicable disease dyads and triads. *Only prevalence of more than 1.5% are presented. CLD – chronic lung disease.

The three NCD dyads with highest prevalence are hypertension + arthritis (11.3%), hypertension + angina (9.8%), and angina + arthritis (9.1%). The three NCD triads with greatest prevalence are hypertension + angina + arthritis (5.2%), hypertension + angina + chronic lung disease (3.4%), and angina + arthritis + chronic lung disease (3.4%) (**Figure 1**).

Tables S3-S4 and Figures S3-S4 in **Online Supplementary Document** show prevalence of each NCD by country; mean outpatient visits, hospitalisation days, and QoL by each NCD; NCD prevalence stratified by age; and prevalence of NCD dyads.

**Figure 2:**
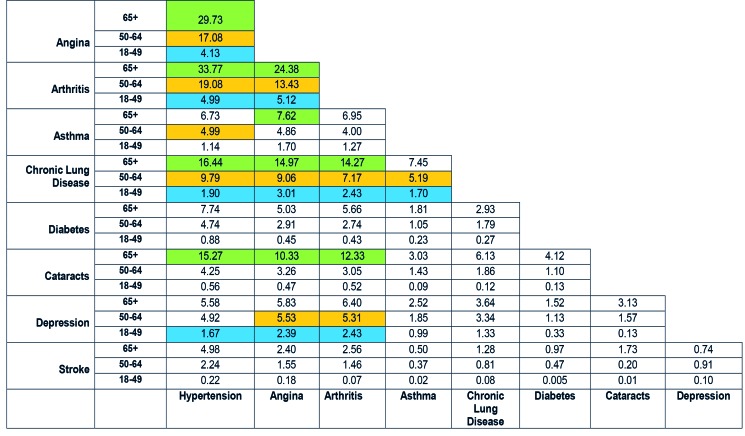
Most prevalent of NCD dyads stratified by age. *Top 10 NCD dyads for each age group: †65+ years = green; 50-64 years = orange; 18-49 years = blue.

Common dyads in elderly subjects included cataracts along with hypertension, angina or arthritis, and also asthma + angina, while the young commonly suffered dyads including depression (depression + hypertension/angina/arthritis) (**Figure 2**). Subjects in the lowest income quintile had asthma + angina/chronic lung disease in the top 10 dyads, while those in the highest wealth quintile had hypertension + cataract/diabetes in the top 10 dyads. Males had chronic lung disease + asthma in their leading dyads, whereas females had hypertension + cataract. Urban-living subjects had hypertension + diabetes/cataract in their top dyads, while rural-living subjects had asthma + angina/chronic lung disease (Figures S5-S8 in **Online Supplementary Document**).

### Health care utilisation

#### Outpatient visits

**Figure 3:**
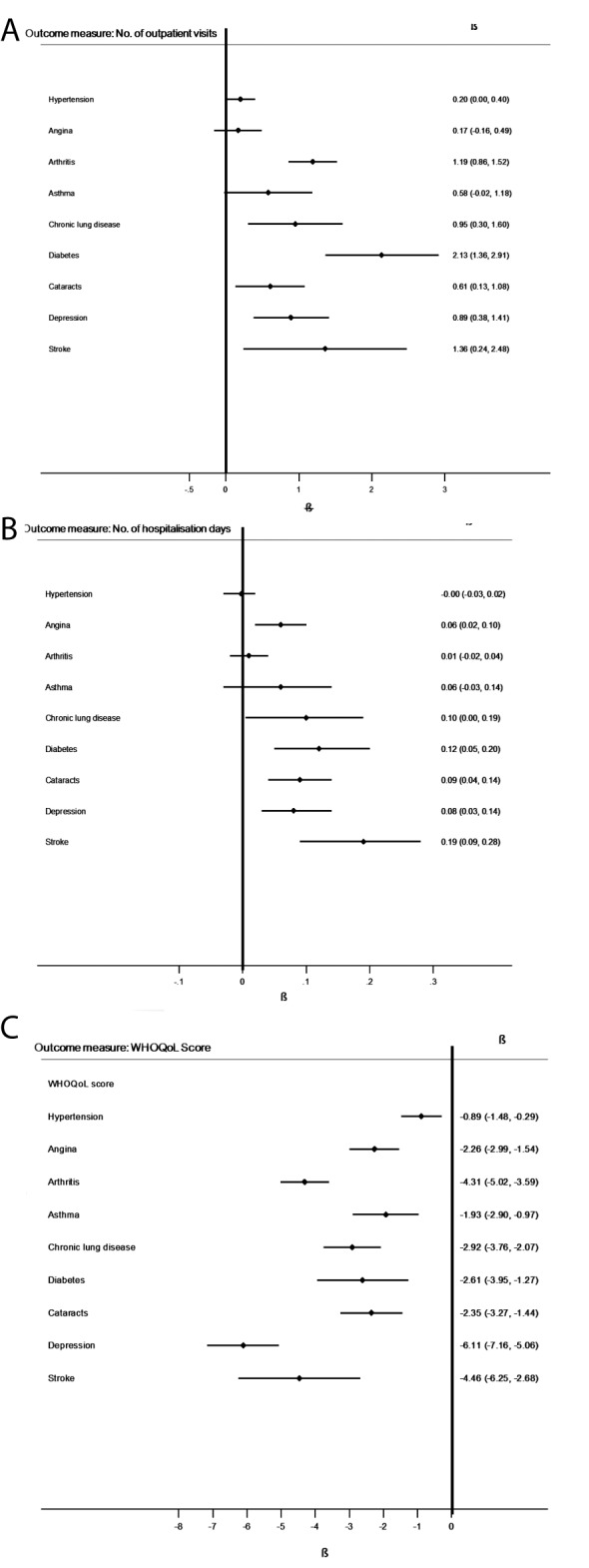
Forest plots of associations between individual NCDs with mean number of outpatient visits, mean number of hospitalisations, and mean WHOQoL score. *Multivariable regression model adjusted for age, gender, marital status, education (primary or less, secondary, tertiary and above), individual’s income quintiles, residence (rural, urban), health insurance (with/without insurance), and country

**Figure 3**, Panel A shows that diabetes, as a single NCD, had the largest effect on increasing the mean number of outpatient visits (β = 2.13, 95%CI = 1.36-2.91, *P* < 0.001).

**Figure 4:**
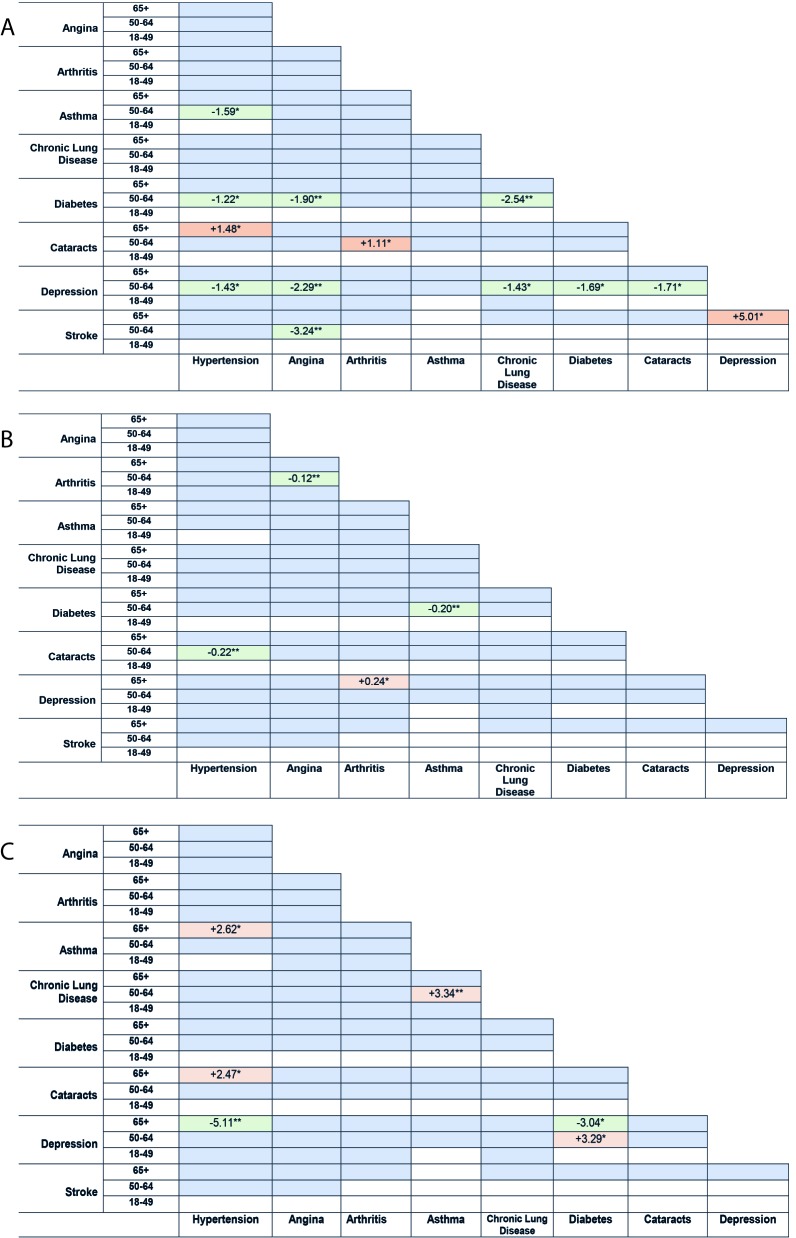
**Panel A.** Association between NCD dyads and health care utilisation (mean number of outpatient visits)‡. **P-value <0.01. *P-value <0.05. *White = sample size, n ≤100. †Green = utilisation-limiting. Orange = Utilisation-increasing. Blue = utilisation-neutral. ‡Multivariable regression model adjusted for age, gender, marital status, education (primary or less, secondary, tertiary and above), individual’s income quintiles, residence (rural, urban), health insurance (with/without insurance), and country. **Panel B.** Association between NCD dyads and health care utilisation (mean number of hospitalisations)‡. **P-value <0.01. *P-value <0.05. †White = sample size, n ≤100. Green = utilisation-limiting. Orange = Utilisation-increasing. Blue = utilisation-neutral. ‡Multivariable regression model adjusted for age, gender, marital status, education (primary or less, secondary, tertiary and above), individual’s income quintiles, residence (rural, urban), health insurance (with/without insurance), and country. **Panel C.** Association between NCD dyads and mean quality of life score‡. **P-value <0.01. *P-value <0.05. †White = sample size, n, ≤100. Green = QoL-limiting. Orange = QoL-increasing. Blue = QoL-neutral. ‡Multivariable regression model adjusted for age, gender, marital status, education (primary or less, secondary, tertiary and above), individual’s income quintiles, residence (rural, urban), health insurance (with/without insurance), and country.

Thirteen dyads were associated with greater or fewer outpatient visits, compared with patients with one NCD each (**Figure 4**, Panel A).

Dyads including depression, diabetes, and hypertension were mostly outpatient visit-limiting. Five dyads of depression in the 50-64 years age-group were utilisation-limiting. Four dyads of diabetes in the 50-64 years age-group were utilisation-limiting. Three dyads of hypertension in the 50-64 years age-group were utilisation-limiting.

#### Hospitalisations

**Figure 3**, Panel B revealed that stroke, as a single NCD, had the largest effect on increasing the mean number of hospitalisations (β = 0.19, 95%CI = 0.09-0.28, *P* < 0.05).

Four dyads were associated with greater or fewer hospitalisations (**Figure 4**, Panel B). The utilisation-increasing depression dyad was depression + arthritis in the 65+ years age-group. Utilisation-limiting dyads were hypertension + cataracts, diabetes + asthma, and angina + arthritis (50-64 years age-group).

### Quality of Life

**Figure 3**, Panel C revealed that depression, as a single NCD, had the largest effect on decreasing mean QoL score (β = -6.11, 95%CI = -7.16 to -5.06, *P* < 0.001).

Five dyads were associated with higher or lower QoL (**Figure 4**, Panel C). Dyads of depression were the most QoL-limiting. In the 65+ years age-group, depression + hypertension and depression + diabetes were QoL-limiting.

## DISCUSSION

### Principal findings

The two NCD dyads with the highest prevalence were hypertension + arthritis and hypertension + angina, while the two NCD triads with the greatest prevalence were hypertension +  angina + arthritis and hypertension +  angina + chronic lung disease. The findings revealed differences in the most common dyads for different age-groups, income levels, gender, and location of residence (urban vs rural).

Diabetes, stroke, and depression (when considered as single diseases) had the largest effect on increasing mean number of outpatient visits, increasing mean number of hospitalisations, and decreasing mean QoL scores, respectively.

We investigated NCD dyads that increased or decreased health care utilisation and QoL, compared with separate patients with one NCD each. Out of the 36 NCD dyads in our study, thirteen, four, and five dyad combinations were associated with higher or lower mean number of outpatient visits, mean number of hospitalisations, or mean QoL scores, respectively, compared with treating separate patients with one NCD each. Dyads of depression were associated with fewer outpatient visits, more hospitalisations, and lower QoL. Dyads including hypertension and diabetes were associated with fewer outpatient visits.

### Comparison of findings with published literature

Our findings that diabetes, stroke, and depression (as single diseases) have the largest effect on increasing outpatient visits, increasing hospitalisations, and decreasing QoL, respectively, are consistent with published studies, most of which have come from high-income countries (HICs) [25-28].

Published studies have shown that depression is an important predictor of poor QoL [28,29]. A study found that depression was the strongest predictor of low QoL for patients with myocardial infarction, compared to factors like living alone, infarction severity, and state anxiety [28]. Our finding that dyads including depression were associated with fewer outpatient visits, more hospitalisations, and lower QoL may suggest that subjects with depression are less likely to receive treatment at the primary care level (general practitioners). These individuals may need more inpatient resources later when depression becomes severe and they experience much lower QoL. Individuals with depression often fail to seek treatment from primary care professionals [30], which may result in more intensive treatment that is needed later – for example, hospitalisations due to relapse and for suicide risk management [31,32].

To date we identified only two other published studies on the effect of NCD combinations on health care utilisation or QOL. A study from the United Kingdom which examined which NCD dyads affected health care costs, found that dyads including depression led to increased cost and dyads including hypertension decreased cost [11]. A study in New Zealand which examined whether the cost of certain NCD dyads were more or less than that expected given the independent costs of each NCD found that neurological and musculoskeletal diseases contributed the largest health system costs [33]. These studies, along with our study, showed that certain NCD combinations substantially increase or decrease different aspects of treatment costs.

### Strengths and limitations

This is the first study on six large MICs that investigated NCD combinations and their effects on health care utilisation and QoL, compared with separate patients with one NCD each. However, there were several limitations of our study. The data were collected in different countries over a few years with possible limitations on quality control. Also, self-assessed and reported medical history may be poorly correlated with medical status, and likely more so in less educated, poor, and rural populations [34]. However earlier studies suggest this may not be a substantial problem as SAGE incorporated measures to minimise these issues [13,35]. SAGE provides the best data available to compare NCDs in MICs and has been used in several previous studies [6,7,13,16,35,36]. The survey methodology included strategies to detect and correct for systematic reporting biases in health interview surveys, such as vignette methods and objective performance tests [14]. Strategies were used to improve data comparability, such as utilising common definitions of concepts, common data collection methods and translations, rigorous sample design, and post-hoc harmonization [14]. As we had no data, we could not include other large MICs such as Indonesia in this study.

Further, outpatient visits were not specific for NCDs and might include unrelated visits for other conditions [6]. Hospitalisations were counted as the number of overnight stays in the hospital that lasted for at least one night, in the last 12 months. This study did not examine the effects of NCD dyads on hospitalisation length. QoL was self-reported using a subjective rating scale. The cross-sectional design limited causal interpretations, and further studies that use prospective designs are needed to examine causality. This study was based on nine NCDs, so further work could examine more conditions, like the large-scale Scotland study with 40 NCDs [37]. Lastly, some apparent associations may be due to chance, so it is important to focus on consistent patterns such as associations with depression, hypertension, and diabetes, rather than isolated findings.

### Policy and research implications

Our study revealed that certain NCDs and NCD dyad combinations were more strongly associated with greater utilisation and lower QoL, with implications for policy and clinical practice [11]. Health policies should improve the accessibility of resources and health care funding to individuals with NCDs with the largest effects on increasing outpatient visits and hospitalisations, and lowering QoL. In addition, our findings on the most prevalent NCD triad and dyad combinations may be useful for the design and management of co-occurring chronic conditions of patients by health care providers, especially when most existing clinical guidelines are for single diseases. There is a growing need for such guidelines to consider how management is different when other comorbidities are present.

Findings about the utilisation-limiting effects of diabetes and hypertension might be explained by the potential for these conditions, which are both risk factors for cardiovascular disease, to be simultaneously managed within one consultation [38]. Health systems could design integrated-care-delivery models for the more prevalent chronic conditions such that patients with NCD combinations that include those conditions would benefit from a more efficient health system with utilisation-limiting outcomes [39].

Our study provided new evidence from MICs that depression may have a greater adverse effect on QoL than physical NCDs, with policy implications for the provision of better psychological and social support for high-risk individuals. Also, the finding that depression was related to fewer outpatient visits but more hospitalisations may suggest that patients with depression were less likely to seek primary care at an early stage, leading to worsening of their depression and other NCDs, and potentially avoidable hospitalisations. This has important implications for improving the identification and treatment of depression at a primary health care level, in addition to optimising the use of inpatient resources.

Future research could focus on how multimorbidity patterns develop and change over the life-course as economic, demographic and epidemiological transitions progress, using prospective designs [38]. This may provide clues about causation and opportunities to prevent the development or worsening of some conditions. Further investigation is needed to explain how dyads interact to produce differential effects on utilisation and QoL.

## Additional material

Online Supplementary Document
