# Supplementary material for: Implications of multimorbidity patterns on health care utilisation and quality of life in middle-income countries: cross-sectional analysis
Source: J Glob Health. 2019 Aug 6;9(2):020413. doi: 10.7189/jogh.09.020413 (PMC6684869; doi:10.7189/jogh.09.020413)
Supplement: Online Supplementary Document [file jogh-09-020413-s001.pdf]

Online Supplementary Documents (OSD)

Figure S1. Flowcharts summarising the process of data cleaning in each country (China, India, Ghana, Russia, Mexico, South Africa)

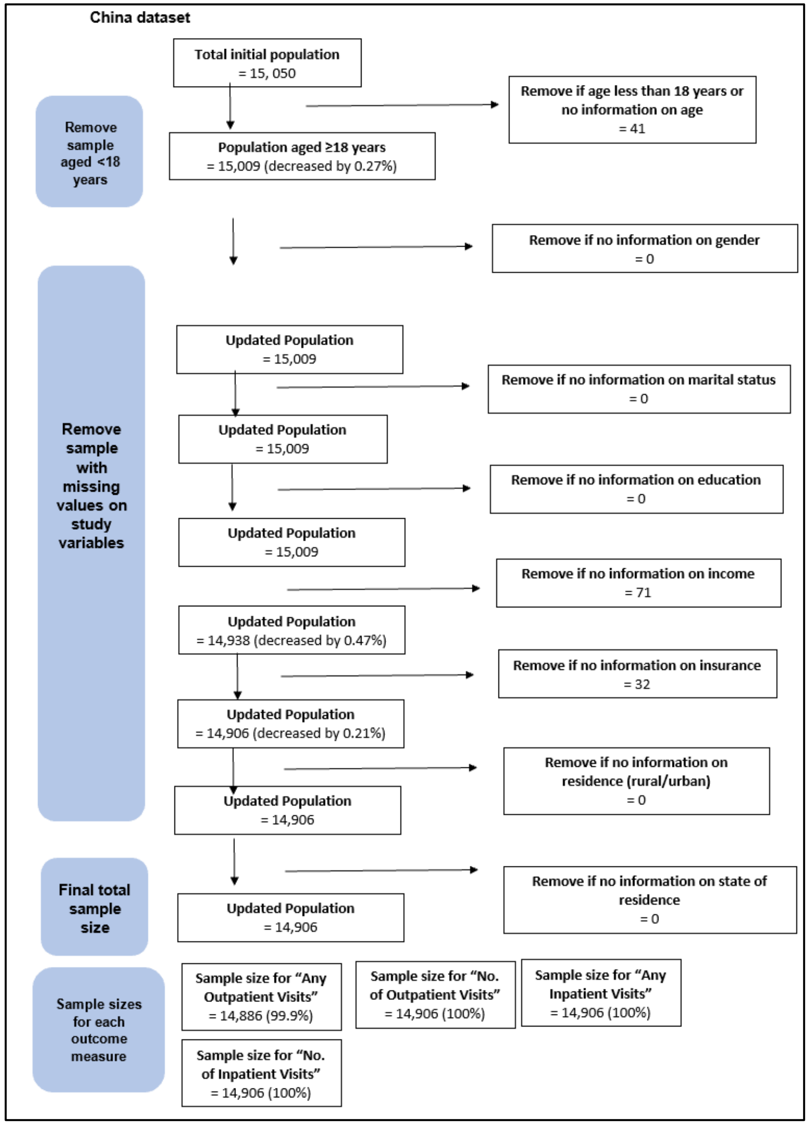

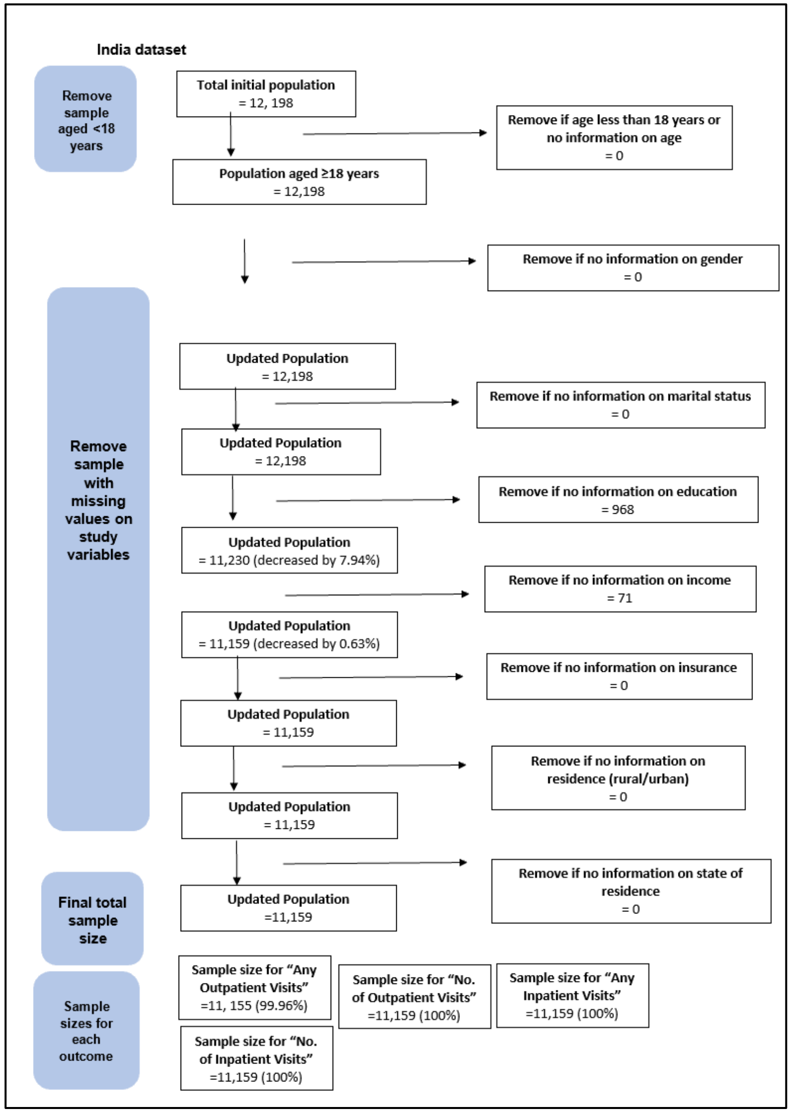

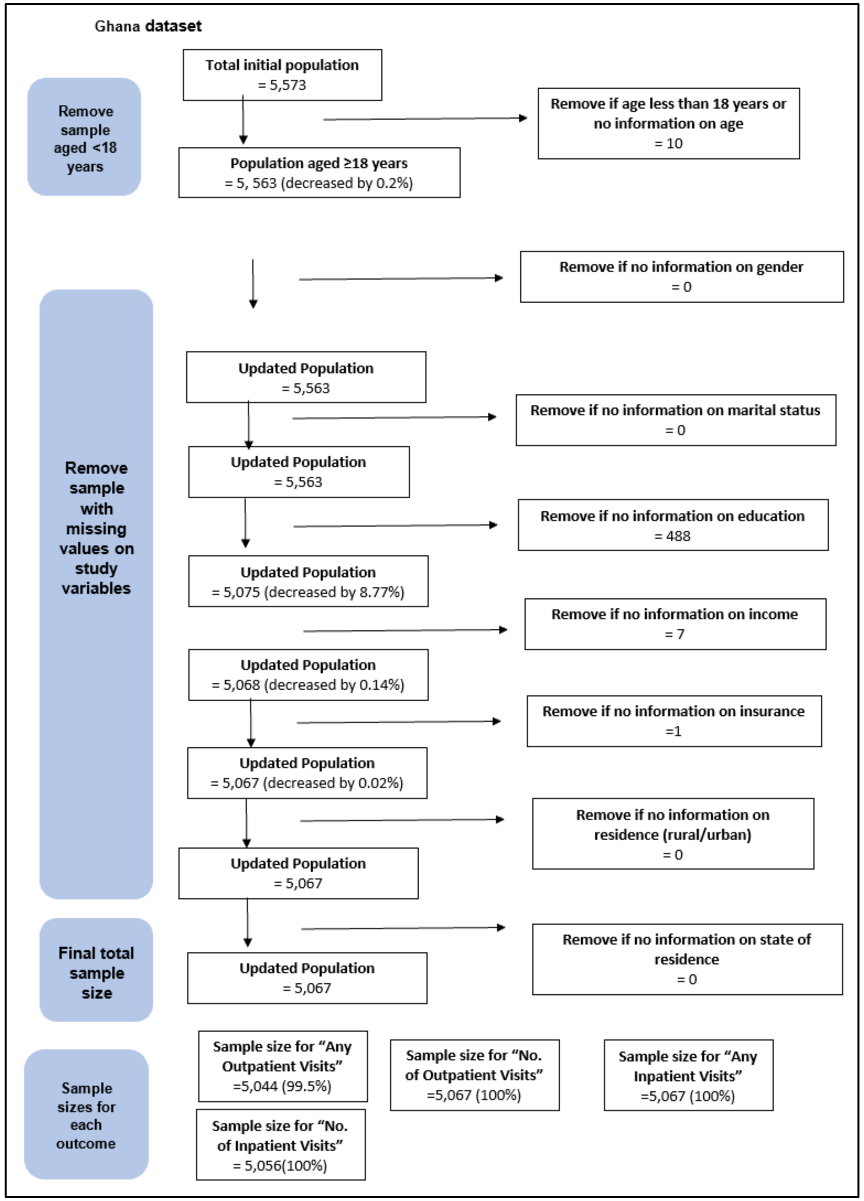
Ghana dataset
Total initial population = 5,573
Remove sample aged <18 years
Remove if age less than 18 years or no information on age = 10
Population aged ≥18 years = 5, 563 (decreased by 0.2%)
Remove if no information on gender = 0
Remove sample with missing values on study variables
Updated Population = 5,563
Remove if no information on marital status = 0
Updated Population = 5,563
Remove if no information on education = 488
Updated Population = 5,075 (decreased by 8.77%)
Remove if no information on income = 7
Updated Population = 5,068 (decreased by 0.14%)
Remove if no information on insurance =1
Updated Population = 5,067 (decreased by 0.02%)
Remove if no information on residence (rural/urban) = 0
Updated Population = 5,067
Final total sample size
Remove if no information on state of residence = 0
Updated Population = 5,067
Sample sizes for each outcome
Sample size for "Any Outpatient Visits" =5,044 (99.5%)
Sample size for "No. of Outpatient Visits" =5,067 (100%)
Sample size for "Any Inpatient Visits" =5,067 (100%)
Sample size for "No. of Inpatient Visits" = 5,056(100%)

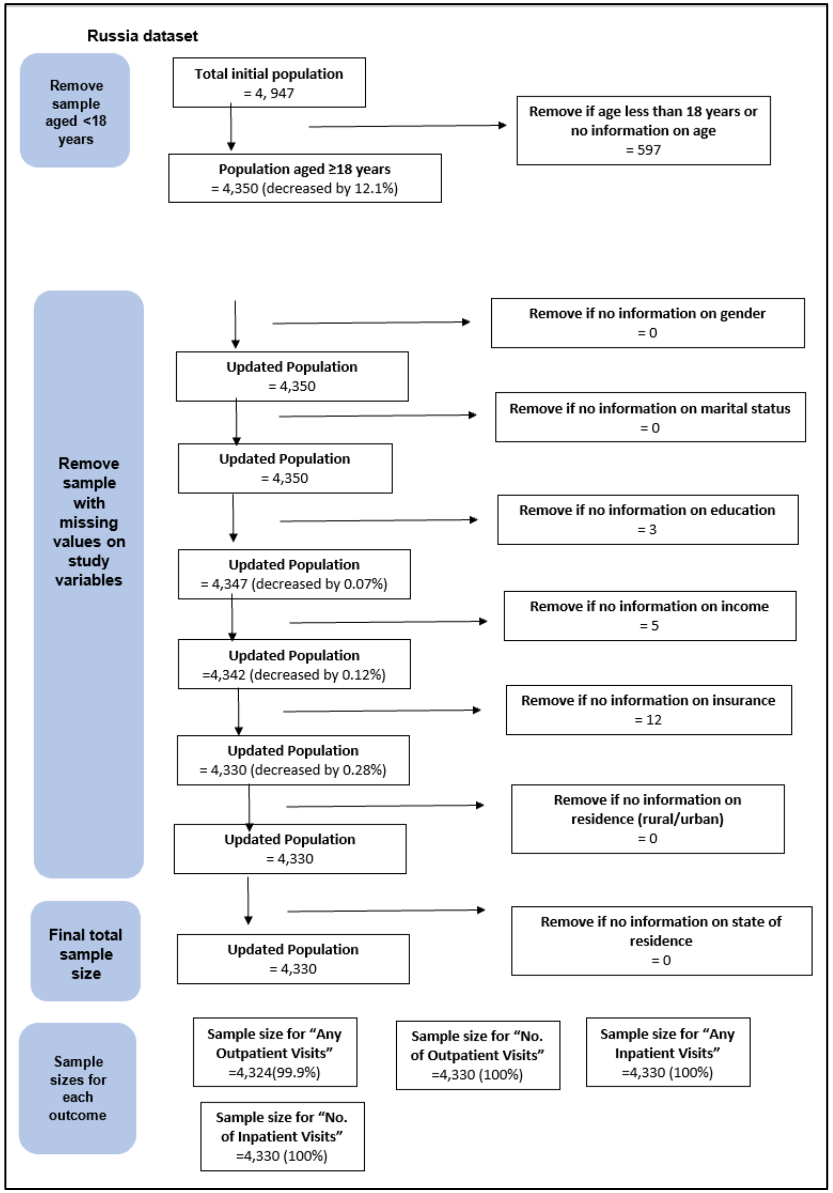
Russia dataset
Total initial population = 4, 947
Remove sample aged <18 years
Remove if age less than 18 years or no information on age = 597
Population aged ≥18 years = 4,350 (decreased by 12.1%)
Remove sample with missing values on study variables
Remove if no information on gender = 0
Updated Population = 4,350
Remove if no information on marital status = 0
Updated Population = 4,350
Remove if no information on education = 3
Updated Population = 4,347 (decreased by 0.07%)
Remove if no information on income = 5
Updated Population =4,342 (decreased by 0.12%)
Remove if no information on insurance = 12
Updated Population = 4,330 (decreased by 0.28%)
Remove if no information on residence (rural/urban) = 0
Updated Population = 4,330
Final total sample size
Remove if no information on state of residence = 0
Updated Population = 4,330
Sample sizes for each outcome
Sample size for "Any Outpatient Visits" =4,324(99.9%)
Sample size for "No. of Outpatient Visits" =4,330 (100%)
Sample size for "Any Inpatient Visits" =4,330 (100%)
Sample size for "No. of Inpatient Visits" =4,330 (100%)

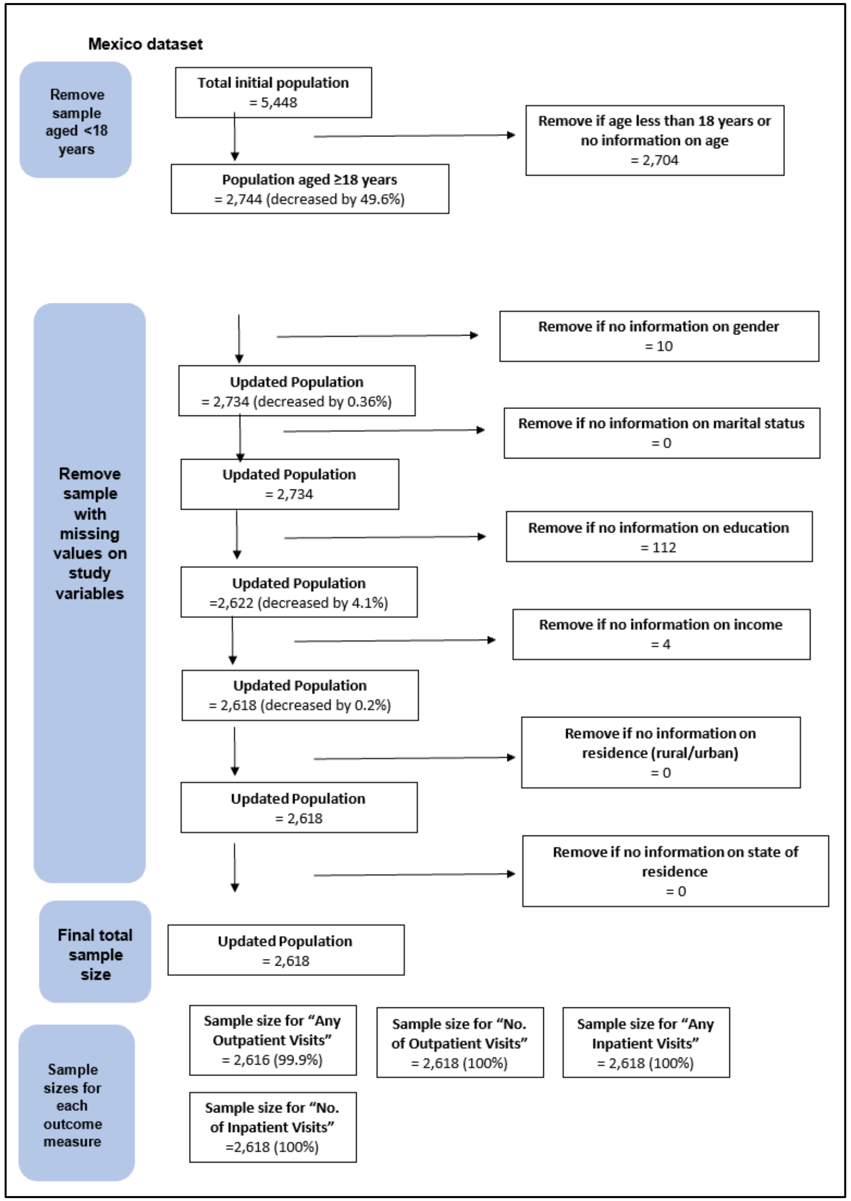
Mexico dataset
Remove sample aged <18 years
Total initial population = 5,448
Remove if age less than 18 years or no information on age = 2,704
Population aged ≥18 years = 2,744 (decreased by 49.6%)
Remove sample with missing values on study variables
Remove if no information on gender = 10
Updated Population = 2,734 (decreased by 0.36%)
Remove if no information on marital status = 0
Updated Population = 2,734
Remove if no information on education = 112
Updated Population =2,622 (decreased by 4.1%)
Remove if no information on income = 4
Updated Population = 2,618 (decreased by 0.2%)
Remove if no information on residence (rural/urban) = 0
Updated Population = 2,618
Remove if no information on state of residence = 0
Final total sample size
Updated Population = 2,618
Sample sizes for each outcome measure
Sample size for "Any Outpatient Visits" = 2,616 (99.9%)
Sample size for "No. of Outpatient Visits" = 2,618 (100%)
Sample size for "Any Inpatient Visits" = 2,618 (100%)
Sample size for "No. of Inpatient Visits" =2,618 (100%)

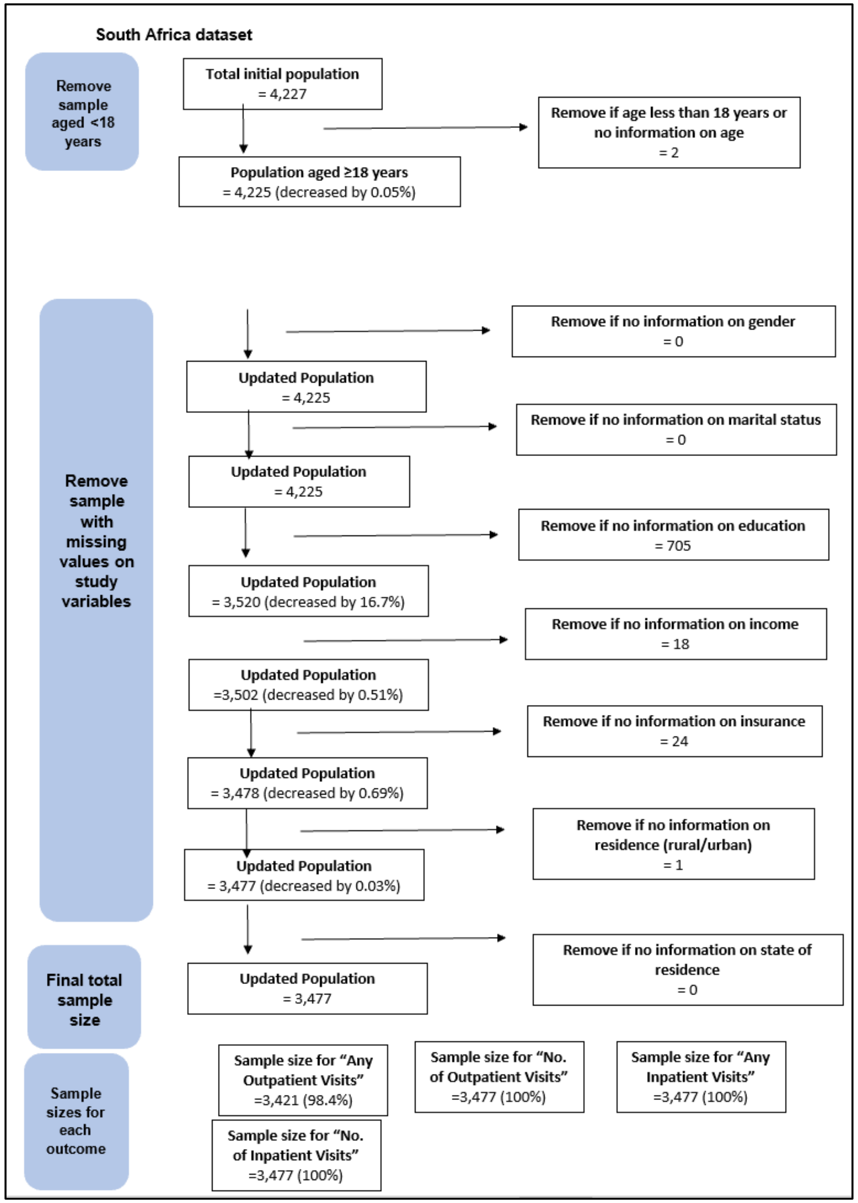
South Africa dataset
Remove sample aged <18 years
Total initial population = 4,227
Remove if age less than 18 years or no information on age = 2
Population aged ≥18 years = 4,225 (decreased by 0.05%)
Remove sample with missing values on study variables
Remove if no information on gender = 0
Updated Population = 4,225
Remove if no information on marital status = 0
Updated Population = 4,225
Remove if no information on education = 705
Updated Population = 3,520 (decreased by 16.7%)
Remove if no information on income = 18
Updated Population =3,502 (decreased by 0.51%)
Remove if no information on insurance = 24
Updated Population = 3,478 (decreased by 0.69%)
Remove if no information on residence (rural/urban) = 1
Updated Population = 3,477 (decreased by 0.03%)
Remove if no information on state of residence = 0
Final total sample size
Updated Population = 3,477
Sample sizes for each outcome
Sample size for "Any Outpatient Visits" =3,421 (98.4%)
Sample size for "No. of Outpatient Visits" =3,477 (100%)
Sample size for "Any Inpatient Visits" =3,477 (100%)
Sample size for "No. of Inpatient Visits" =3,477 (100%)

Table S1. Algorithms used to ascertain NCD presence

NCDs	Self-reported diagnosis	Biomarkers/Symptom-based Assessment	Algorithm
Hypertension	Have you ever been diagnosed with high blood pressure?	Take average of three blood pressure readings. High blood pressure is defined as systolic blood pressure≥140mmHg and diastolic blood pressure ≥90mmHg.	High blood pressure is defined as systolic blood pressure≥140mmHg and diastolic blood pressure ≥90mmHg.
Stroke	Have you ever been told by a health professional that you have had a stroke?	No symptom-based assessment	No symptom-based assessment
Diabetes	Have you ever been diagnosed with diabetes?	No symptom-based assessment	No symptom-based assessment
Cataract	In the last 5 years, were you diagnosed with a cataract in one or both of your eyes?	No symptom-based assessment	No symptom-based assessment
Arthritis	Have you ever been diagnosed with/told you have arthritis (a disease of the joints, or by other names rheumatism or osteoarthritis)?	**Qn 1:** During the last 12 months, have you experienced pain, aching, stiffness or swelling in or around the joints (like arms, hands, legs or feet) which were not related to an injury and lasted for more than a month?<br>a. Yes<br>b. No<br><br>**Qn 2:** During the last 12 months, have you experienced stiffness in the joint in the morning after getting up from bed, or after a long rest of the joint without movement?<br>a. Yes<br>b. No<br><br>**Qn 3:** How long did the stiffness last?<br>a. about 30mins or less<br>b. more than 30 mins<br><br>**Qn 4:** Did this stiffness go away after exercise or movement in the joint?<br>a. Yes<br>b. No	Arthritis=<br><br>Option ‘a’ to **both** question 1 and question 2.<br><br>OR<br><br>Option ‘a’ to **both** question 3 and question 4.
Angina	Have you ever been diagnosed with angina or angina pectoris?	**Qn 1:** During the last 12 months, have you experienced any pain or discomfort in your chest when you walk uphill or hurry?<br>a. Yes<br>b. No<br><br>**Qn 2:** During the last 12 months, have you experienced any pain or discomfort in your chest when you walk at an ordinary pace on level ground?<br>a. Yes<br>b. No	Angina=<br>Option ‘a’ for question 1<br><br>OR<br><br>Option ‘a’ for question 2<br><br>OR

		**Qn 3:** What do you do if you get the pain or discomfort when you are walking?<br>a. stop or slow down<br>b. carry on after taking a pain-relieving medicine that dissolves in your mouth<br>c. carry on walking<br><br>**Qn 4:** If you stand still, what happens to the pain or discomfort?<br>a. relieved<br>b. not relieved<br><br>**Qn 5:** Will you show me where you usually experience the pain or discomfort?<br>a. (6 & 11), or (7 & 8).	Option 'a' for **both** question 3 and question 4.<br><br>OR<br><br>Option 'a' for question 5
Asthma	Have you ever been diagnosed with asthma?	**Qn 1:** Attacks of wheezing or whistling breathing?<br>a. Yes<br>b. No<br><br>**Qn 2:** Attack of wheezing that came on after you stopped exercising or some other physical activity?<br>a. Yes<br>b. No<br><br>**Qn 3:** A feeling of tightness in your chest?<br>a. Yes<br>b. No<br><br>**Qn 4:** Have you woken up with a feeling of tightness in your chest in the morning or any other time?<br>a. Yes<br>b. No<br><br>**Qn 5:** Have you had an attack of shortness of breath that came on without obvious cause when you were not exercising or doing some physical work?<br>a. Yes<br>b. No	Asthma=<br>Option 'a' for Qn 1<br><br>**OR**<br><br>Option 'a' for **all** questions from question 2 to question 5.
Chronic lung disease	Have you ever been diagnosed with chronic lung disease (emphysema, bronchitis, COPD)?	**Qn 1:** During the last 12 months, have you experienced any shortness of breath at rest? (while awake)<br>a. Yes<br>b. No<br><br>**Qn 2:** During the last 12 months, have you experienced any coughing or wheezing for ten minutes or more at a time?<br>a. Yes<br>b. No<br><br>**Qn 3:** During the last 12 months, have you experienced any coughing up sputum or phlegm for most days of the month for at least 3 months?<br>a. Yes<br>b. No	Chronic lung disease=<br>Option 'a' for Qn 1<br><br>**OR**<br><br>Option 'a' for **both** question 2 and question 3

Depression	Have you ever been diagnosed with depression?	**Qn 1:** During the last 12 months, have you had a period lasting several days when you felt sad, empty or depressed?<br>a. Yes<br>b. No<br><br>**Qn 2:** During the last 12 months, have you had a period lasting several days when you lost interest in most things you usually enjoy such as personal relationships, work or hobbies/recreation?<br>a. Yes<br>b. No<br><br>**Qn 3:** During the last 12 months, have you had a period lasting several days when you have been feeling your energy decreased or that you are tired all the time?<br>a. Yes<br>b. No<br><br>Qn 4: Was this period [of sadness/loss of interest/low energy] for more than 2 weeks?<br>a. Yes<br>b. No<br><br>Qn 5: Was this period [of sadness/loss of interest/low energy] most of the day, nearly every day?<br>a. Yes<br>b. No<br><br>Qn 6: During this period, did you lose your appetite?<br>a. Yes<br>b. No<br><br>Qn 7: Did you notice any slowing down in your thinking?<br>a. Yes<br>b. No<br><br>Qn 8: Did you notice any problems falling asleep?<br>a. Yes<br>b. No<br><br>Qn 9: Did you notice any problems waking up too early?<br>a. Yes<br>b. No<br><br>Qn 10: During this period, did you have any difficulties concentrating: for example, listening to others, working, watching TV, listening to the radio?<br>a. Yes<br>b. No<br><br>Qn 11: Did you notice any slowing down in your moving around?<br>a. Yes<br>b. No	**Group A:**<br>Score=1 if option 'a' to question 1<br>Score=1 if option 'a' question 2<br><br>Score=1 if option 'a' to question 3<br><br>**Addition of score= score for Group A**<br><br>**Group B:**<br>Score=1 if option 'a' to either question 8 or question 9<br><br>Score=1 if option 'a' to either question 7 or question 10<br><br>Score=1 if option 'a' to either question 11 or question 13<br><br>Score=1 if option 'a' to either question 14 or question 15<br><br>Score=1 if option 'a' to either question 17 or question 18<br><br>**Addition of score= score for Group B**<br><br>**Group C:**<br>Score=1 if option 'a' to question 6<br><br>**Group D:**<br>Score=1 if option 'a' to question 12<br><br>**Group E:**<br>Score for Group B + Score for Group C + Score for Group D<br><br>**Group F:**<br>Score=1 if option 'a' to question 4<br><br>**Group G:**<br>If Score for Group A ≥2, **AND** Score for Group F is =1, add the score for Score for Group A + Score for Group E

		Qn 12: During this period, did you feel anxious and worried most days?<br>a. Yes<br>b. No<br><br>Qn 13: During this period, were you so restless or jittery nearly every day that you paced up and down and couldn't sit still?<br>a. Yes<br>b. No<br><br>Qn 14: During this period, did you feel negative about yourself or like you had lost confidence?<br>a. Yes<br>b. No<br><br>Qn 15: Did you frequently feel hopeless- that there was no way to improve things?<br>a. Yes<br>b. No<br><br>Qn 16: During this period, did your interest in sex decrease?<br>a. Yes<br>b. No<br><br>Qn 17: Did you think of death, or wish you were dead?<br>a. Yes<br>b. No<br><br>Qn 18: During this period, did you ever try to end your life?<br>a. Yes<br>b. No	**Depression=**<br>**Score for Group G ≥4**

Table S2. Sample characteristics of the population of China, India, Ghana, Russia, Mexico, and South Africa

	China	India	Ghana	Russia	Mexico	South Africa	Pooled
**Total (n)**	14,906	11,159	5,067	4,330	2,618	3,477	41,557
**Gender (%)**							
Male	46.7	38.74	52.65	35.64	38.20	39.72	43.01
Female	53.3	61.26	47.35	64.36	61.80	60.28	56.99
**Marital Status (%)**							
Not married	16.75	22.34	41.70	46.26	41.29	55.88	29.19
Married	83.25	77.66	58.30	53.74	58.71	44.12	70.81
**Age Group (%)**							
18-29	1.44	14.27	2.53	2.26	2.18	2.04	5.20
30-39	3.41	14.75	5.94	3.39	6.49	2.59	6.89
40-49	6.05	12.54	7.20	3.95	7.30	3.36	7.57
50-59	38.70	26.16	33.04	33.76	16.23	40.47	32.86
60-69	26.46	19.92	23.60	24.57	34.91	29.59	24.95
70+	23.94	12.37	27.69	32.06	32.89	21.94	22.53
**Multimorbidity (%)**							
**0 NCDs**	61.68	74.75	56.80	50.21	68.43	48.33	64.00
**1 NCD**	24.47	10.47	26.02	11.13	14.76	33.95	17.00
**2 or more NCDs**	13.85	14.78	17.18	38.66	16.81	17.72	19.00
**Mean number of NCDs**	1.03	1.41	1.23	1.88	1.36	1.40	1.11
**Education Level (%)**							
No schooling	23.93	45.18	50.74	0.95	17.07	24.04	30.09
Primary or lower	35.66	25.79	23.03	9.01	59.43	47.66	31.20
Secondary	21.27	12.47	5.51	18.15	10.62	14.75	15.44
Tertiary or higher	19.14	16.55	20.72	71.89	12.87	13.55	23.27
**Wealth Quintile %)**							
Q1 (lowest)	19.03	17.85	19.44	17.78	20.59	20.13	18.82
Q2	19.82	19.23	19.62	19.40	20.55	20.19	19.67
Q3	20.01	19.14	19.76	19.98	18.56	19.64	19.62
Q4	20.66	21.02	20.70	20.35	20.66	20.07	20.68
Q5 (highest)	20.48	22.75	20.49	22.49	19.63	19.96	21.20
**Location (%)**							
Rural	50.87	74.53	59.05	24.32	26.70	33.62	52.49
Urban	49.13	25.47	40.95	75.68	73.30	66.38	47.51
**Insurance (%)**							
No insurance	12.97	95.91	63.77	0.48	-	82.17	48.14
With insurance (mandatory/voluntary)	87.03	4.09	36.23	99.52	-	17.83	51.86

Table S3. Prevalence of each non-communicable disease (NCD) by country

	NCD Prevalence (%)								
	**Hypertension**	**Angina**	**Arthritis**	**Asthma**	**Chronic Lung Disease**	**Diabetes**	**Cataract**	**Depression**	**Stroke**
**China**	55.06	7.70	18.16	2.27	5.60	2.87	2.57	1.56	0.89
**India**	32.08	24.15	22.21	7.83	10.04	3.18	6.05	12.41	1.05
**Ghana**	55.18	12.67	20.74	3.09	2.30	2.15	2.27	5.13	1.38
**Russia**	66.47	33.88	35.14	7.48	21.76	5.98	9.39	8.47	4.36
**Mexico**	61.19	10.42	13.46	3.58	14.03	9.78	3.88	12.44	1.75
**South Africa**	75.44	8.04	15.81	4.60	4.75	3.41	2.02	6.61	1.58

Table S4. Outcomes measures for each individual non-communicable disease (NCD) using pooled data

Outcome Measure	Non-communicable disease								
	**Hypertension**	**Angina**	**Arthritis**	**Asthma**	**Chronic Lung Disease**	**Diabetes**	**Cataract**	**Depression**	**Stroke**
**Mean no. of outpatient visits**	2.41	3.10	3.41	3.80	3.62	4.90	3.64	3.80	4.10
**Mean no. of hospitalisations**	0.14	0.21	0.17	0.27	0.26	0.31	0.26	0.25	0.36
**Mean QoL Score**	71.70	67.86	67.62	66.29	66.58	67.52	67.07	64.18	65.57

Figure S2. Single non-communicable disease (NCD) prevalence stratified by age using pooled data

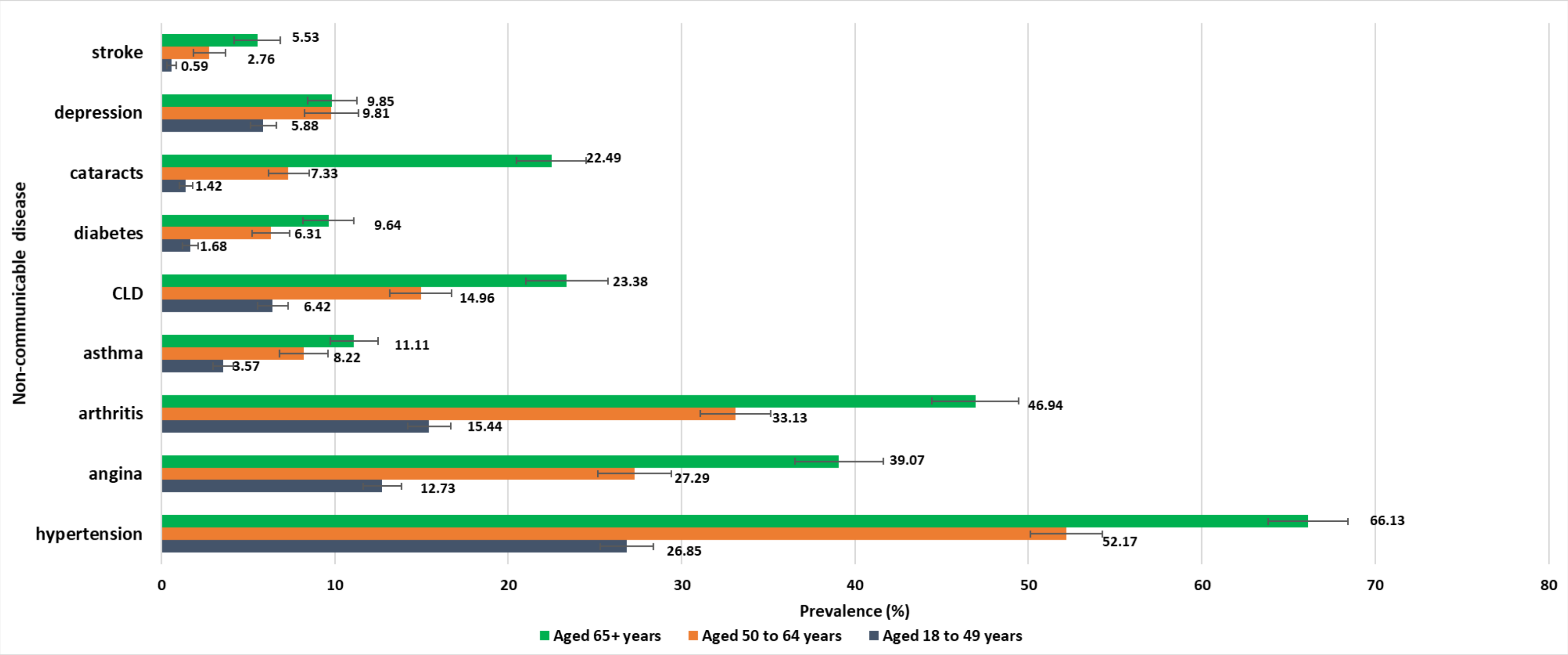

Figure S3. Multimorbidity prevalence using pooled data

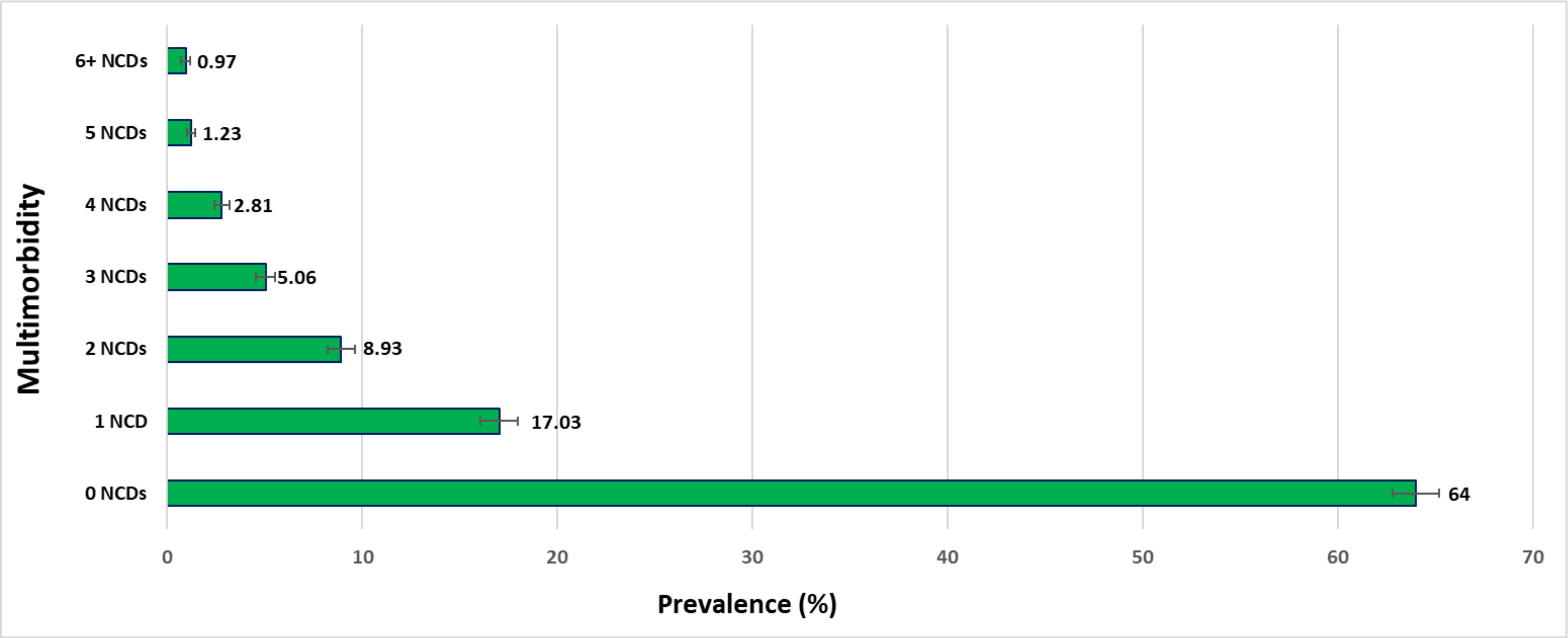

Figure S4. Multimorbidity prevalence stratified by age using pooled data

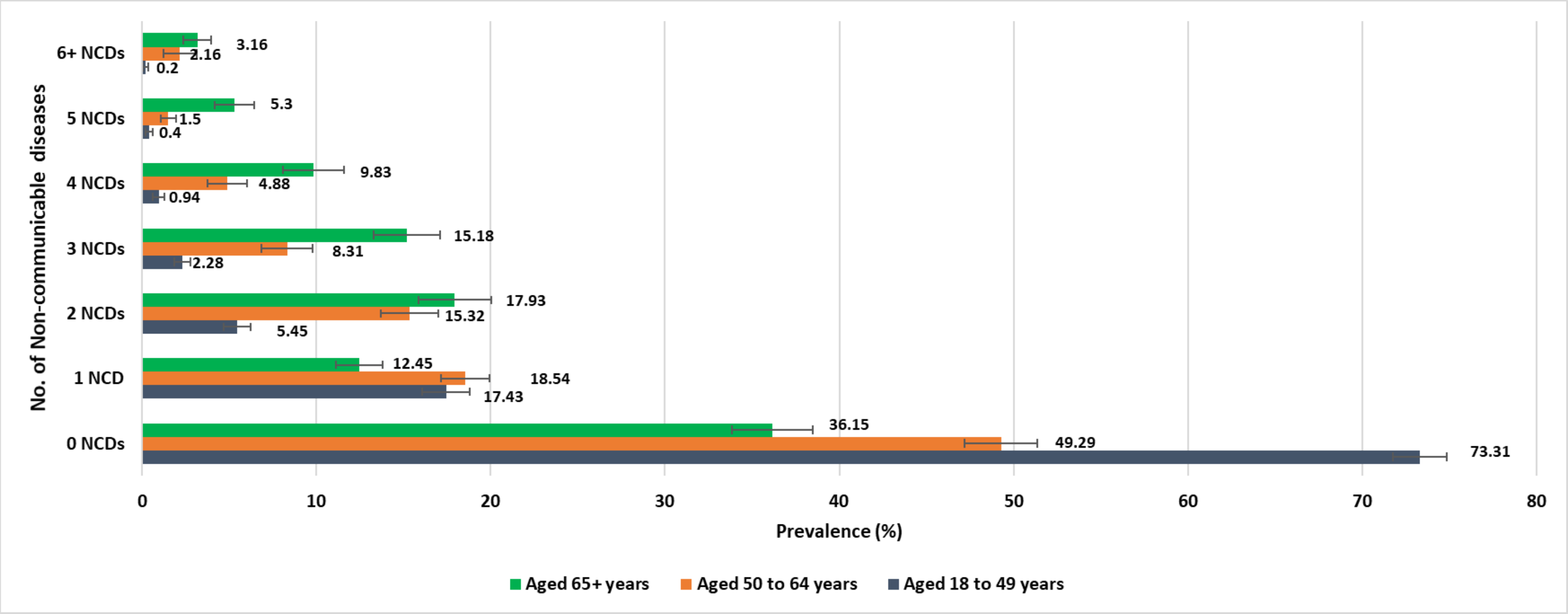

Figure S5. Prevalence of non-communicable disease (NCD) dyads using pooled data

Prevalence:
>3%=Green
2-3%=Yellow
1-2%=Orange

	Hypertension	Angina	Arthritis	Asthma	Chronic Lung Disease	Diabetes	Cataracts	Depression
**Angina**	9.84							
**Arthritis**	11.32	9.13						
**Asthma**	2.59	3.05	2.51					
**Chronic Lung Disease**	5.24	5.68	4.82	3.10				
**Diabetes**	2.48	1.50	1.53	0.59	0.90			
**Cataracts**	3.10	2.24	2.48	0.71	1.19	0.81		
**Depression**	2.79	3.43	3.48	1.35	2.01	0.64	0.79	
**Stroke**	1.20	0.73	0.65	0.15	0.37	0.22	0.26	0.34

Figure S6. Most prevalent non-communicable disease (NCD) dyads stratified by wealth quintiles using pooled data

Top 10 most common NCD pairs for the highest and lowest wealth quintiles:
Q1= Green; Q5= Orange

**Angina**	**Q1 (lowest)**	10.50							
	**Q5 (highest)**	8.24							
**Arthritis**	**Q1**	12.82	12.76						
	**Q5**	8.87	6.87						
**Asthma**	**Q1**	2.87	4.58	3.95					
	**Q5**	1.83	1.74	1.58					
**Chronic Lung Disease**	**Q1**	6.20	8.27	7.30	4.54				
	**Q5**	4.27	4.29	3.67	1.86				
**Diabetes**	**Q1**	1.45	1.23	1.25	0.87	0.92			
	**Q5**	2.98	1.48	1.73	0.30	0.86			
**Cataracts**	**Q1**	2.67	2.44	3.06	0.93	1.78	0.70		
	**Q5**	2.74	1.70	2.08	0.62	0.79	0.75		
**Depression**	**Q1**	3.15	4.99	4.18	2.86	3.45	0.72	0.91	
	**Q5**	2.49	2.98	2.76	0.81	2.08	0.73	0.57	
**Stroke**	**Q1**	1.04	0.66	0.77	0.14	0.23	0.04	0.15	0.15
	**Q5**	1.33	0.86	0.64	0.07	0.53	0.38	0.16	0.47
		**Hypertension**	**Angina**	**Arthritis**	**Asthma**	**Chronic Lung Disease**	**Diabetes**	**Cataracts**	**Depression**

Figure S7. Most prevalent non-communicable disease (NCD) dyads stratified by gender using pooled data

Top 10 most common NCD dyads by gender:
Male= Green; Female= Orange

Angina	Male	7.90							
	Female	11.71							
Arthritis	Male	8.69	6.80						
	Female	13.86	11.39						
Asthma	Male	2.64	2.95	2.37					
	Female	2.54	3.15	2.64					
Chronic Lung Disease	Male	5.06	5.68	4.26	3.44				
	Female	5.42	5.67	5.37	2.76				
Diabetes	Male	2.36	1.41	1.26	0.73	1.07			
	Female	2.60	1.58	1.79	0.44	0.73			
Cataracts	Male	2.64	1.90	2.02	0.67	1.08	0.77		
	Female	3.56	2.56	2.92	0.76	1.29	0.85		
Depression	Male	2.48	2.90	3.26	1.44	1.96	0.74	0.64	
	Female	3.10	3.94	3.70	1.27	2.06	0.54	0.93	
Stroke	Male	1.35	0.76	0.66	0.17	0.46	0.31	0.27	0.39
	Female	1.06	0.69	0.64	0.13	0.29	0.13	0.25	0.28
		Hypertension	Angina	Arthritis	Asthma	Chronic Lung Disease	Diabetes	Cataracts	Depression

Figure S8. Most prevalent non-communicable disease (NCD) dyads stratified by residence (urban versus rural) using pooled data

Top 10 most common NCD dyads by residence:
Urban= Green; Rural= Orange

**Angina**	**Urban**	11.80							
	**Rural**	8.19							
**Arthritis**	**Urban**	12.44	9.29						
	**Rural**	10.37	9.00						
**Asthma**	**Urban**	2.90	2.69	2.20					
	**Rural**	2.32	3.36	2.76					
**Chronic Lung Disease**	**Urban**	5.91	5.64	4.79	2.88				
	**Rural**	4.68	5.71	4.85	3.28				
**Diabetes**	**Urban**	3.45	1.89	1.90	0.65	1.14			
	**Rural**	1.67	1.17	1.23	0.53	0.69			
**Cataracts**	**Urban**	4.27	2.80	2.95	0.87	1.62	1.24		
	**Rural**	2.12	1.76	2.07	0.58	0.82	0.46		
**Depression**	**Urban**	2.88	3.27	2.96	1.09	1.96	0.80	0.83	
	**Rural**	2.72	3.57	3.92	1.57	2.05	0.50	0.75	
**Stroke**	**Urban**	1.73	1.05	0.93	0.22	0.61	0.43	0.44	0.54
	**Rural**	0.76	0.45	0.42	0.09	0.17	0.04	0.11	0.17
		**Hypertension**	**Angina**	**Arthritis**	**Asthma**	**Chronic Lung Disease**	**Diabetes**	**Cataracts**	**Depression**
